# Apraclonidine Is Better Than Cocaine for Detection of Horner Syndrome

**DOI:** 10.3389/fneur.2019.00055

**Published:** 2019-01-31

**Authors:** Fion Bremner

**Affiliations:** Department of Neuro-Ophthalmology, National Hospital for Neurology and Neurosurgery, London, United Kingdom

**Keywords:** Horner syndrome, apraclonidine, cocaine, pharmacological testing, pupillometry, test sensitivity

## Abstract

**Background:** In suspected cases of Horner syndrome pharmacological confirmation is often required before embarking on further investigations. There are two drugs currently used for this purpose that are commercially available for topical administration: cocaine (2–10%) and apraclonidine (0.5–1.0%).

**Aims:** To evaluate and compare the effects of both drugs in normal eyes and eyes with Horner syndrome

**Methods:** This is a retrospective study looking at the outcome of 660 consecutive pharmacological tests with these two drugs in one tertiary referral center over 14 years. Eyes were categorized as “normal” or “Horner syndrome” based on *non-pharmacological* criteria (pupillometric and clinical evidence). Pupil diameters in the dark and in bright light were measured by pupillometry before and 40 min after administration of the test drug (either 4% cocaine or 0.5% apraclonidine).

**Results:** Cocaine dilated the normal pupil (measured in bright light: mean +2.1 mm, range −0.4 to +3.9 mm; 95% lower limit +0.5 mm); the extent of this response was not significantly affected by patient age or pupil size, but was 50% less in brown eyes compared with blue or green eyes, and 20% less if the measurements were made in the dark. In eyes with Horner syndrome cocaine had significantly less mydriatic effect (mean +0.7 mm, range −0.7 to +2.9 mm). Apraclonidine constricted the normal pupil (measured in the dark: mean −0.4 mm, range −1.3 to +0.8 mm; 95% upper limit +0.1 mm); eye color made no difference but the response was significantly greater in younger patients and larger pupils and significantly less if measured in bright lighting conditions. In eyes with Horner syndrome apraclonidine dilated the pupil (mean +0.6, range −0.4 to +2.3 mm). Applying the 95% limits identified from my normative data, I estimate the sensitivity of each drug test for detection of Horner syndrome at 40% for cocaine (criterion for abnormal: mydriasis ≤0.5 mm when measured in the dark) compared with 93% for apraclonidine (criterion for abnormal: mydriasis ≥0.1 mm when measured in the dark).

**Conclusions:** Apraclonidine is a more sensitive test than cocaine for detection of Horner syndrome, and should be adopted as the new gold standard in routine clinical practice. However, caution is needed when using this drug within hours of a suspected sympathetic lesion, or in infants under 1 year of age.

## Introduction

The clinical signs associated with disruption to the sympathetic nerve supply to the eye have been known for almost 150 years since their first description by Horner [(1); see also (2)]. The clinical importance of recognizing Horner syndrome (HS) lies not in its effects on the eye (oculosympathetic denervation has no impact on sight or on the health of the eye) but in the potential seriousness of the underlying cause: in some cases HS may be the first and only sign of life-threatening conditions such as tumors or dissection of the internal carotid artery (3–5). Clinicians must therefore remain alert to the sometimes subtle signs of HS and investigate accordingly.

**Figure 1:**
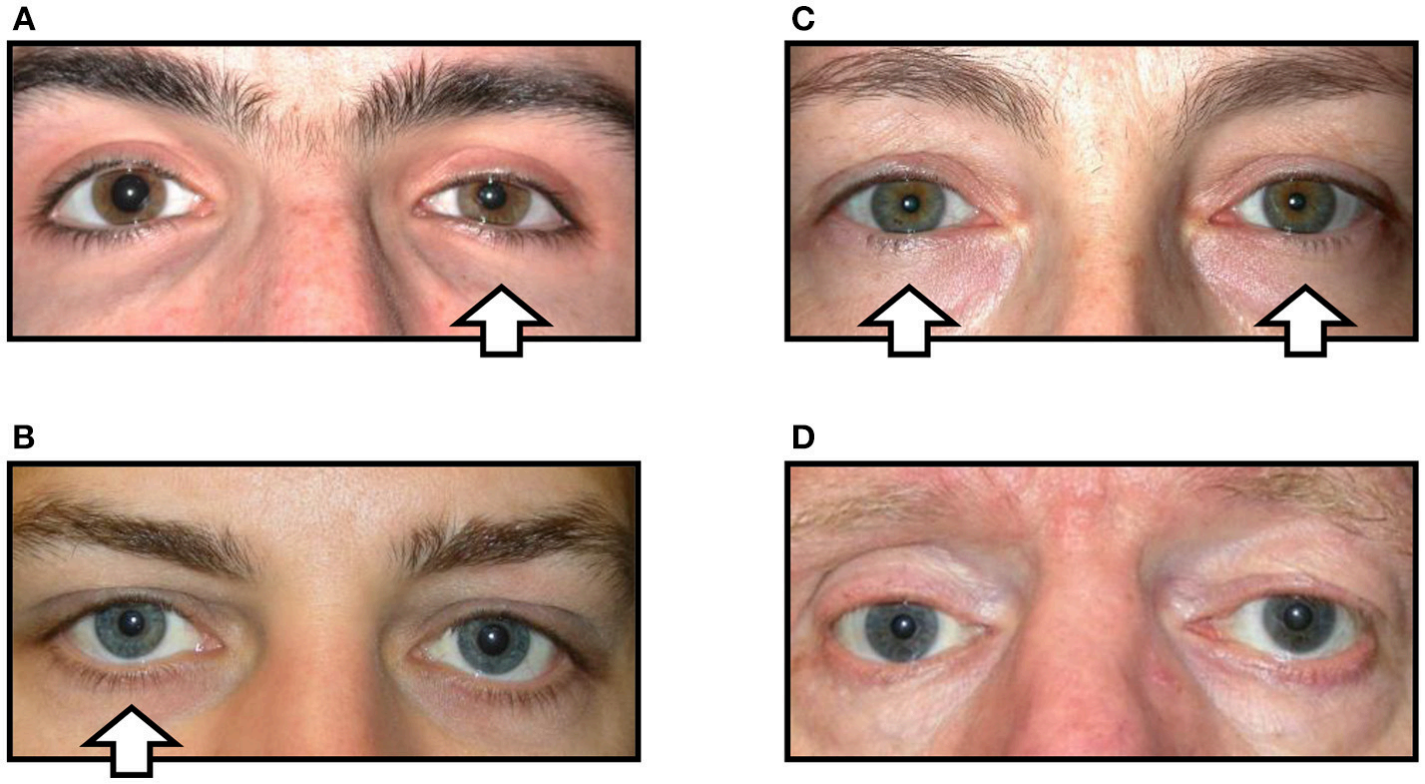
Variations in the clinical signs associated with Horner syndrome (arrows indicate side of the oculosympathetic paresis). **(A)** “Complete” Horner syndrome, with relative ptosis of the upper lid, elevation of the lower lid, miosis of the pupil, and injection of the conjunctiva. **(B)** “Incomplete” Horner syndrome, with relative miosis but no ptosis. **(C)** “Bilateral” Horner syndrome, with no lid or pupil asymmetry. **(D)** “Pseudo-Horner syndrome”: lid asymmetry is associated with right-sided enophthalmos and hypoglobus following an old orbital floor fracture; the anisocoria is physiological.

In his original description Horner merely noted relative miosis of the ipsilateral pupil and ptosis of the upper lid, but subsequent reports have added further details to this clinical phenotype according to the types of sympathetic fiber affected by the lesion. When the sympathetic *pupillomotor* fibers are affected, the ipsilateral pupil has a smaller resting diameter, dilates poorly in dim lighting conditions and slowly (“redilation lag”) after cessation of a transient light stimulus. Involvement of the *motor* fibers innervating Mueller's muscle in the upper lid cause mild ptosis (1–2 mm) that persists in downgaze, and in the lower lid involvement of the equivalent fibers causes the lid margin to elevate by 1–2 mm giving rise to a narrowed palpebral aperture (“pseudo-enophthalmos”). Disruption to the accompanying *vasomotor* fibers leads to relative hypotony, mild injection and chemosis of the conjunctiva, and interference with the ability of the facial skin to “flush” in response to thermal, emotional or gustatory stimulation. Impairment of the *sudomotor* fibers causes loss of sweating so that the ipsilateral skin is drier compared with the unaffected side. The typical appearance of HS is shown in Figure 1A.

However, in clinical practice it is common to encounter patients in whom the signs of HS are more difficult to detect. For example, any lesion that only disrupts some of the sympathetic fibers may cause a *partial* HS [e.g., miosis but no ptosis, or vice versa; see Figure 1B; (6)]. In other cases the underlying pathology may give rise to “diffuse” sympathetic neuropathy rather than any focal lesion; in these cases there is typically *bilateral* HS and the clinical signs of the oculosympathetic paresis are masked because there is no resulting asymmetry of pupil size or lid position [see Figure 1C; (7)]. In both of these circumstances the diagnosis of HS is easily missed by the clinician (false negative). Conversely, patients may present with miosis and/or ptosis that is not caused by a lesion to the ocular sympathetic supply [*pseudo-HS* (8)]. An example is shown in Figure 1D; this patient was referred to me for investigation of what was presumed to be right HS, but in fact his anisocoria is physiological (note that anisocoria is greater in the dark than in the light both when it is physiological and when it is caused by HS) and the lid asymmetry is accompanied by mild enophthalmos and related to a past (and long-forgotten) orbital floor fracture. In these false positive cases an incorrect clinical inference of HS may lead to unnecessary further investigations and distress to the patient.

**Figure 2:**
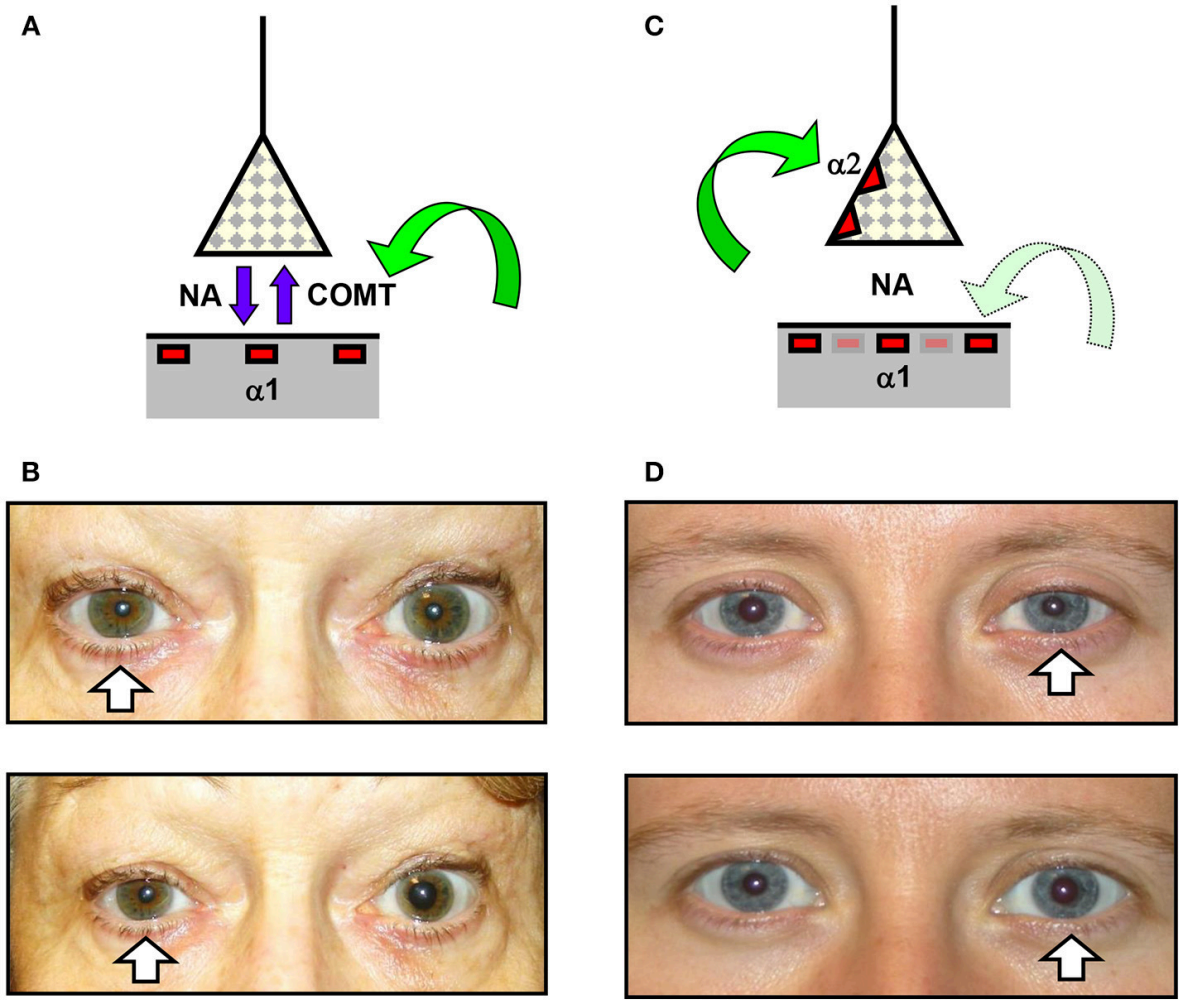
Effects of cocaine and apraclonidine on the pupil. **(A,C)** Schematic diagrams of the neuro-effector junction in the iris dilator muscle, showing the sympathetic nerve ending releasing noradrenaline (NA) to bind with alpha-1 adrenoceptors on the muscle membrane. **(A)** Cocaine blocks the enzyme cyclo-oxygenase methyl transferase (COMT) which provides the active re-uptake mechanism terminating the action of noradrenaline, and so the concentration of noradrenaline rises and the normal pupil dilates. **(C)** Apraclonidine is an adrenergic agonist with greater affinity for the presynaptic alpha-2 receptors (which inhibit noradrenaline release) than the post-synaptic alpha-1 receptors, so in a normal pupil the alpha-2 effect predominates and the pupil mioses. **(B,D)** Photographs of the pupils before (upper) and after (lower) cocaine **(B)** or apraclonidine **(D)** eye drops in two patients with unilateral Horner syndrome (arrows). Cocaine is seen to increase the degree of anisocoria, whereas apraclonidine causes the anisocoria to reverse.

Given the unreliability of the clinical signs in some cases of oculosympathetic paresis, it is often necessary to perform additional pharmacological testing to confirm the suspicion of HS before embarking on further investigations. Two commercially available drugs have generally been used for this purpose, cocaine and apraclonidine (I have chosen not to consider dilute phenylephrine in this study since it is not generally available as a proprietary formulation). Cocaine has been used for over 50 years [see (9)] and is still considered by many to be the “gold standard” test for HS [see (10)]. It blocks the active reuptake of noradrenaline by the sympathetic nerve endings, thereby increasing neurostransmitter availability and dilating the normal pupil; in contrast the drug has less mydriatic effect in HS (because there is less of the neurotransmitter “lying around”) so the test is considered positive if the drug *increases* the degree of resting anisocoria (Figures 2A,B). More recently it has been reported that apraclonidine can be used to diagnose HS (11, 12). This adrenergic agonist predominantly activates alpha-2 receptors—which in the eye are found on the presynaptic sympathetic nerve endings and inhibit release of noradrenaline, causing miosis of the normal pupil; however in HS sympathetic denervation leads to an upregulation of alpha-1 receptors on the post-junctional dilator muscle fibers so that the weaker alpha-1 effects of apraclonidine now predominate, dilating the pupil and *reversing* the anisocoria (see Figures 2C,D).

The question arises as to which of these two drugs provides the more reliable diagnostic test for HS (10)? In both cases there are small case series published suggesting good test sensitivity (13, 14) but there are also reports of false positive and false negative test results with both drugs (13, 15–17), confirming that neither test is perfect. There has only been one published “head-to-head” comparison (18) which in a small series of just 10 patients showed that both drugs reliably identified the HS. In this retrospective study I have evaluated the effects of these two drugs in a large number of “normal” eyes and in eyes for which there is compelling *non-pharmacological* evidence of HS. Despite using a standardized protocol for conducting the tests I have found a wide variation in drug effect in normal eyes, and further analysis has identified some of the more important confounders influencing the test result. In eyes where I felt there was definite non-pharmacological evidence of oculosympathetic paresis, my data suggests that apraclonidine provides a more sensitive test for HS than cocaine, and on that basis I recommend that this drug should now be considered the “gold standard.” However, there are some circumstances in which cocaine should be used instead, and these are also discussed.

## Materials and Methods

In this retrospective study I have looked at the results of cocaine or apraclonidine testing in all adult patients undergoing pupillometry in a tertiary referral center between 2004 and 2018. The review of these data formed part of a Clinical Service Evaluation (CSE) registered with the Queen Square Quality & Safety Team; they have approved my use of these data and confirmed that this study adheres to their local Information Governance Policy as well as the tenets of the Declaration of Helsinki.

Using either my own custom-built pupillometer or the Procyon P3000D proprietary device (both devices use video cameras running at 25 Hz with spatial resolution of 0.03 mm), all patients underwent a standard battery of pupillometric evaluations, including measurements of the resting pupil diameter (averaged over 3 s) in complete darkness and in bright light (room lights “full on”), measurements relating to the average reflex constriction of the pupil to three repetitions of a standard 1 s white light stimulus (on average the intensity of this light was sufficient to constrict the pupil by 30%) and measurements of the mydriatic response to a sudden loud noise (“startle response”) [see (19, 20) for details of my methodology]. Pupillometric confirmation of a sympathetic paresis (“Horner”) is implied if the pupil shows a reduced capacity to dilate in darkness, delayed redilation after cessation of a transient light stimulus [this “T3/4” measurement is considered delayed if it lies outside the 95% upper limit of normal relative to the reflex constriction amplitude based on my own normative database; (7, 21)] and an absent startle response [see (20) for full details of the measurement, calculation and interpretation of these pupillometric parameters]. Patients also had a full ocular and neuro-ophthalmic assessment by an experienced clinician (FB). Where relevant, and depending on the clinical context, some patients went on to have further investigations including autonomic function tests, blood tests (e.g., serology for ganglionic nicotinic acetylcholine receptor antibodies) and imaging studies, providing in many cases further clinical evidence of a lesion or pathology likely to cause Horner syndrome. Based on these pupillometry measurements and the results of any subsequent investigations, the tested eyes have been categorized as “normal” (i.e., showing no evidence of oculosympathetic paresis), “Horner” (i.e., strong pupillometric or other *non-pharmacological* evidence to suggest a sympathetic lesion), or “unclear” (cases where the evidence was incomplete or conflicting). I have excluded from any further analysis all of these “unclear” cases (diagnosis not established; constituted approximately 3% of all cases) and also any patients with ocular disease or on ocular medications that might interfere with this evaluation (invalid data; total number unknown as not added to database, but likely to be small).

In all cases pharmacological testing for Horner syndrome was routinely performed as part of the initial pupillometric evaluation using either 4% cocaine or 0.5% apraclonidine eye drops (drug used depended only on availability and convenience and was not selected according to any clinical criteria). The standard protocol throughout this period has been to measure the pupil diameter both in complete darkness and with the room lights “full on” before and 40 min after administration of the test drug. Note was also made of the iris color, which was photographed and categorized as brown, green or blue. In a small number of cases both drugs were evaluated, allowing an interval of at least 48 h “wash-out time” between tests.

Standard statistical approaches have been used to estimate the 95% upper and lower limits to the pupil response of “normal” eyes to these drugs. Linear regression models were then used to assess the influence on this drug effect of patient age, pupil size, eye color, and the lighting conditions (dark or light). The responses of pupils in eyes with Horner syndrome were compared with those in normal eyes using either unpaired *t*-tests (if data normally distributed) or the Mann-Whitney rank sum test (if normality test failed). In cases of unilateral Horner syndrome, paired *t*-tests were used to compare the drug responses in the affected and unaffected eyes. The 95% limits of the drug effects as identified in my normative data were used to categorize the *pharmacological* test results as “normal” or “Horner,” and these were compared in a 2 × 2 contingency table with the categorization based on *non-pharmacological* test results (pupillometric and/or clinical) to provide estimates of the sensitivity and specificity of each of the two drugs for detecting a sympathetic lesion. McNemar's chi-squared test was used to estimate the concordance of test results in the small number of cases where both tests were performed. All statistical tests were performed using SigmaStat (Systatsoftware Ltd., version 3.5).

## Results

### Normal Eyes

Over the study period, drug testing was performed in 493 eyes judged to be “normal” (i.e., where there was no clinical or pupillometric evidence of HS and no other ocular disease or exposure to medication that might affect the test result). The age and gender of these patients is shown in Table 1.

**Table 1:** Demographics of patients with normal pupils and patients with Horner syndrome undergoing drug testing.

		**4% cocaine**		**0.5% apraclonidine**	
		**Normal**	**Horner**	**Normal**	**Horner**
**Number of eyes**		182	95	311	72
Age	Mean	46	50	43	53
	Range	18–80	8–82	14–82	23–73
Gender	Male	36%	42%	29%	39%
	Female	64%	58%	71%	61%
Iris color	Brown	21%		21%	
	Green	28%		31%	
	Blue	51%		48%	

**Figure 3:**
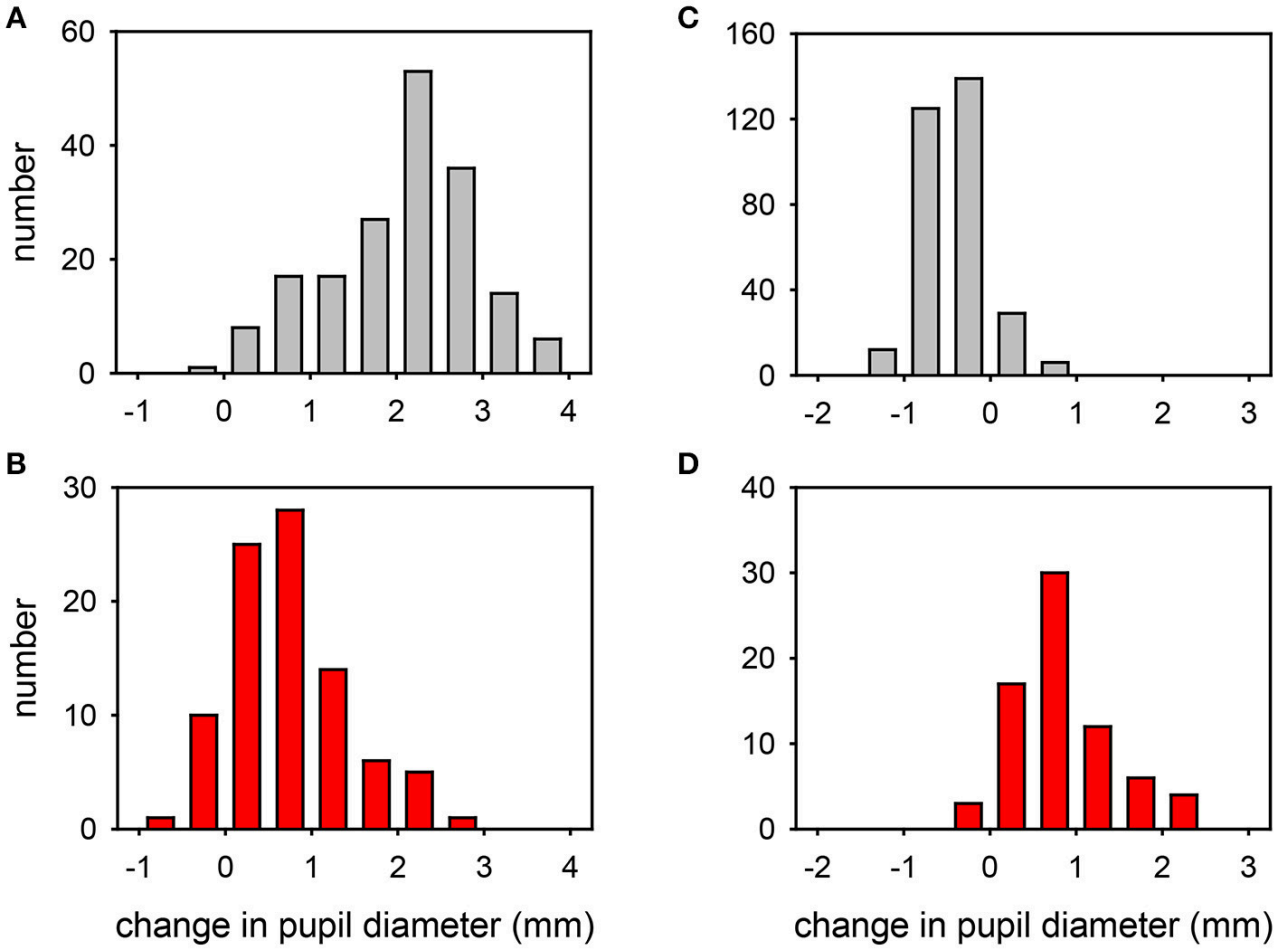
Frequency distribution plots of the change in pupil diameter induced by cocaine **(A,B)** or apraclonidine **(C,D)** in normal eyes **(A,C)** and eyes with Horner syndrome **(B,D)**. Cocaine measurements were made in bright light, apraclonidine measurements were made in the dark.

The effects of exposing these normal eyes to one drop of either 4% cocaine (pupils measured in the light) or 0.5% apraclonidine (pupils measured in the dark) are summarized in Table 2, and the frequency histograms of these drug effects are shown in Figures 3A,C, respectively. Cocaine on average caused a +2.06 mm increase in pupil diameter (equivalent to a relative 37% increase in pupil size), but the drug effect was highly variable in different eyes, ranging from −0.36 to +3.90 mm (−10 to +121%), and the distribution of these measurements failed standard normality testing (Kolmogarov-Smirnov). In contrast, apraclonidine on average caused a 0.44 mm decrease in pupil diameter (equivalent to a relative 7% decrease in pupil size), with measured effects ranging from −1.3 to +0.8 mm (−20 to +16%) and the distribution of results also failing normality testing.

**Table 2:** Effect of drugs on the size of the pupil in normal eyes and in eyes with Horner syndrome.

		**4% cocaine**		**0.5% apraclonidine**	
		**Normal**	**Horner**	**Normal**	**Horner**
**Number of eyes**		182	95	311	72
Drug effect	Mean	+2.06	+0.72	−0.44	+0.73
	SD	0.85	0.71	0.35	0.60
	Range	−0.36 to +3.90	−0.68 to +2.94	−1.3 to +0.8	−0.36 to +2.25

*Measurements (in mm) were made in the light for cocaine testing and in the dark for apraclonidine testing. “+” Indicates an increase in pupil size, “−” indicates a decrease*.

**Figure 4:**
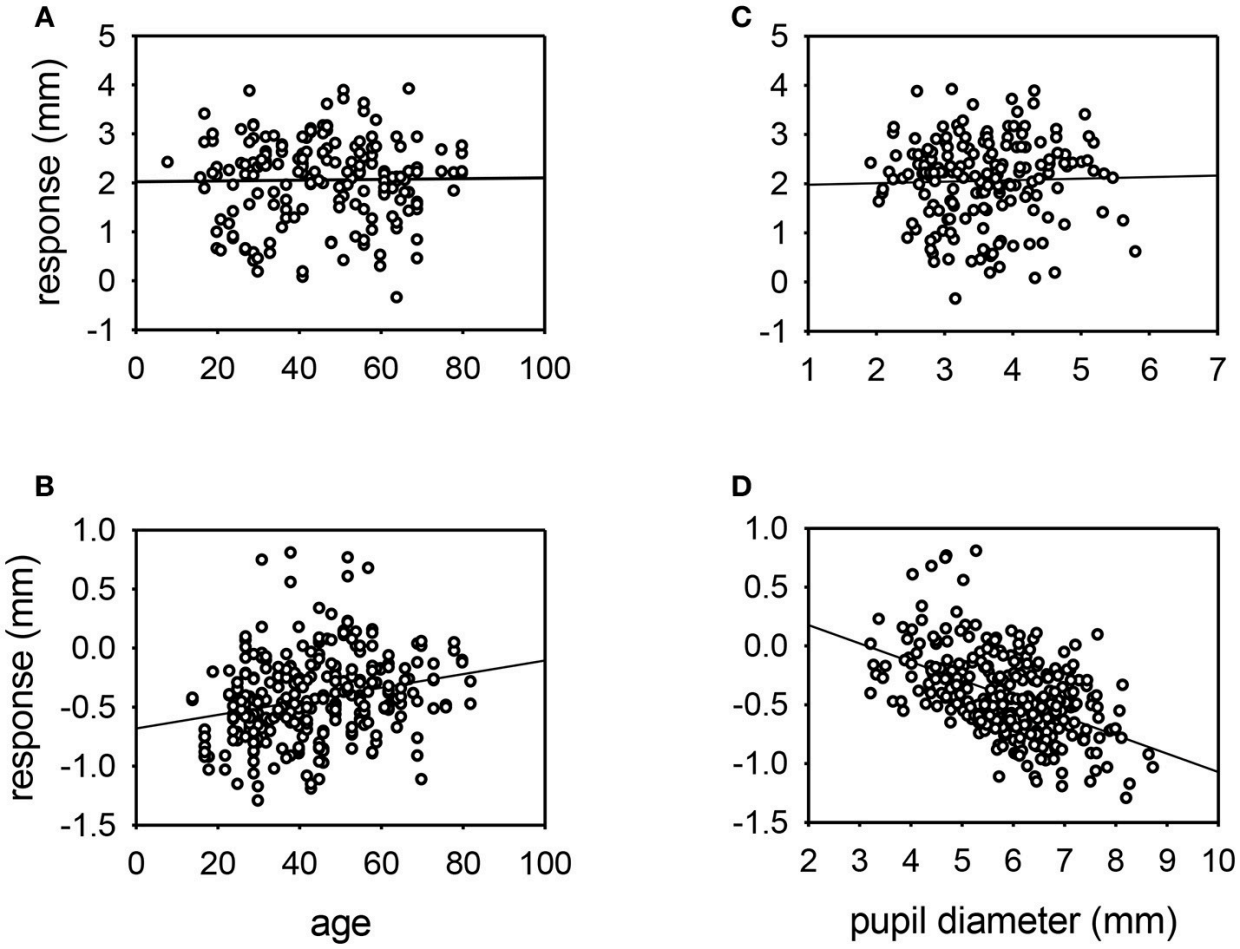
Scatter plots of the response of the pupil to cocaine **(A,C)** or apraclonidine **(B,D)** with respect to age **(A,B)** or resting pupil size **(C,D)**. Linear regression lines are shown. No significant relationships were demonstrated for cocaine responses, but apraclonidine had significantly greater miotic effect in younger patients and in eyes with larger pupils (*P* < 0.001).

**Figure 5:**
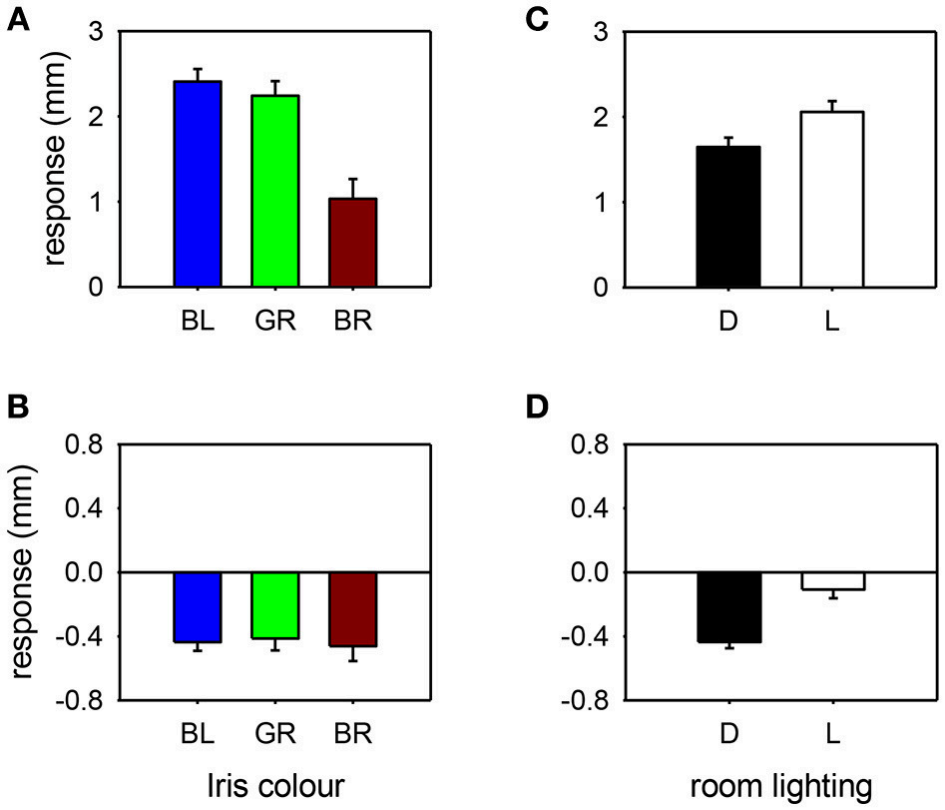
Pupil response to cocaine **(A,C)** or apraclonidine **(B,D)** according to iris color [**(A,B)**: BL, blue; GR, green; BR, brown] or room lighting conditions [**(C,D)**: D, measured in the dark; L, measured in bright light]. Cocaine had significantly greater effect on blue and green eyes compared with brown eyes, and greater effect when measurements were made in bright light compared with darkness. Apraclonidine had similar effects regardless of iris color, but significantly more effect when measurements were made in the dark compared with bright light.

The influence of various parameters on the pupillary response to drug testing has been evaluated (see Figures 4, 5). Linear regression analysis revealed no significant relationships between the age of the patient (*P* = 0.201) or the size of the pupil (*P* = 0.696) and the mydriatic response to cocaine drops. In contrast, the miotic effect of apraclonidine was significantly greater in younger patients (*P* < 0.001) and in bigger pupils (*P* < 0.001). Iris color was found to significantly influence the size of the response of pupils to cocaine: the mydriatic effect in brown eyes was less than half that measured in green or blue eyes (unpaired *t*-tests: *P* < 0.001). However, iris color did not seem to influence the response to apraclonidine which had similar miotic effects in brown, green, and blue eyes. The lighting conditions in which the pupil measurements were made significantly affected the size of the response to both drugs. Cocaine had an average mydriatic effect of +2.06 mm in the light, but only +1.65 mm in the dark (*P* < 0.001). Apraclonidine had an average miotic effect of −0.44 mm in the dark (7%), but only −0.12 mm (2%) in the light.

### Eyes With Horner Syndrome

Over the same study period, drug testing has also been performed on 167 eyes in which there was clear clinical and pupillometric evidence of oculosympathetic paresis (HS). In 50 patients the HS was bilateral and associated with various causes of generalized autonomic failure (e.g., autoimmune autonomic ganglionopathy, pure autonomic failure, dopamine beta-hydroxylase deficiency etc.), whereas in 67 cases the HS was unilateral and either associated with focal lesions (e.g., internal carotid artery dissection, tumors, surgery) or idiopathic. The age and gender of these patients is shown in Table 1.

The effects of exposing these HS eyes to one drop of either 4% cocaine (pupils measured in the light) or 0.5% apraclonidine (pupils measured in the dark) are summarized in Table 2, and the frequency histograms of these drug effects are shown in Figures 3B,D, respectively. Cocaine had some mydriatic effect in most HS eyes, but the mean response (+0.71 mm, 21%) was significantly smaller than the effect in normal eyes (difference = 1.3 mm, *P* < 0.001). Similar results were obtained in the subset of patients in whom the HS was unilateral: cocaine produced a mydriatic effect that was on average 1.1 mm less in the affected eye than in the unaffected “control” eye (paired *t*-test: *P* < 0.001), and as a result the observed degree of anisocoria was increased by cocaine by an average of 1.1 mm (paired *t*-test: *P* < 0.001).

**Figure 6:**
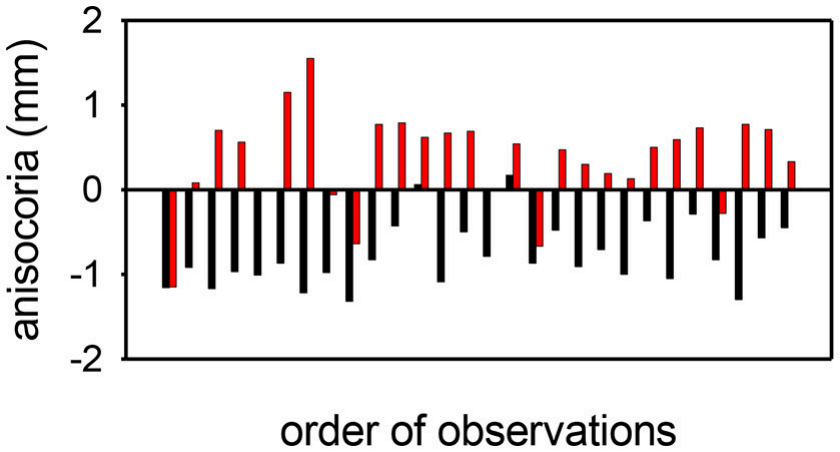
Anisocoria measurements (affected eye—unaffected eye) before (black) and after (red) apraclonidine in 28 consecutive cases of unilateral Horner syndrome. In most cases (21/28) the anisocoria reverses, but in a small number of cases the anisocoria is in the same direction after as before apraclonidine, giving a sensitivity for the test using this criterion of 75%. In all but 2 cases, apraclonidine produced an abnormal degree of mydriasis (≥0.1 mm) in the pupil of the affected eye, giving a sensitivity for the test using this criterion of 93%.

Apraclonidine generally had the opposite effect in HS eyes compared with normal eyes. On average, apraclonidine induced a +0.73 mm (+19%) mydriasis in HS eyes, which was significantly different from the miotic effect seen in normal eyes (normality test failed, so Mann Whitney Rank Sum test used: difference = 1.2 mm, *P* < 0.001). Similar results were obtained in the subset of patients in whom the HS was unilateral: apraclonidine produced a mydriatic effect that was on average 1.1 mm greater in the affected eye than in the unaffected “control” eye (paired *t*-test: *P* < 0.001), and as a result the observed degree of anisocoria was increased by apraclonidine by an average of 1.1 mm (paired *t*-test: *P* < 0.001). Figure 6 shows the observed degree of anisocoria (pupil diameter in affected eye—pupil diameter in unaffected eye) before and after apraclonidine in each of these successive 28 cases of unilateral HS. In general, the affected eye had a smaller pupil before apraclonidine and a larger pupil after apraclonidine, but this “reversal in anisocoria” was only seen in 21 of 28 cases.

How accurate are these drug tests in detecting oculosympathetic paresis? To answer this question I used the data on drug effects in normal eyes to define the 95% limits, outside of which the pupil response would be deemed abnormal (a “positive” test result). Since for both drugs the distribution of these data failed normality testing, I used the rank order of responses to define these limits. For cocaine, my data suggest that any response (measured in bright light) ≤+0.50 mm is abnormal; using this definition, the cocaine test was positive in 36 cases of HS, but negative in 54 cases giving an estimated sensitivity for this test of 40%. When the pupil measurements were instead made in the dark, the 95% lower limit of the normal response to cocaine was +0.14 mm giving an even lower estimation of the sensitivity of the test as a means of diagnosing HS (21%). In unilateral cases of HS an alternative definition has been proposed by another research group (13) who suggested that the cocaine test can be considered positive if the anisocoria after exposure to the drug ≥0.8 mm; using this definition, 29 of the 38 patients with unilateral HS in this study had a positive cocaine test result, giving a sensitivity in these unilateral cases of 76%.

With apraclonidine, my data suggest that any response (measured in the dark) ≥+0.10 mm is abnormal; using this definition, the apraclonidine test was positive in 67 cases of HS, and negative in only 5 cases giving an estimated sensitivity for this test of 93%. When the pupil measurements were made instead in bright light, the 95% lower limit of the normal response to apraclonidine is +0.54 mm giving a slightly lower estimation of the sensitivity of the test as a means of diagnosing HS (76%).

In a small number of cases (*N* = 33) both cocaine and apraclonidine tests were performed in the same eye (allowing at least a 48 h washout period between testing). Applying the above definitions of when a test result is considered negative (normal) or positive (HS), concordant results were found in 22/33 cases. Using McNemar's test this level of concordance gave a chi-squared value of 0.82 confirming no significant difference between the results obtained with these two different drugs (*P* = 0.37).

## Discussion

Both cocaine and apraclonidine are currently used in clinical practice for diagnosing HS, but there are no large studies investigating their effects on the normal pupil. In this retrospective study I found that on average the normal pupil dilates by 2.1 mm with 4% cocaine and constricts by 0.4 mm with 0.5% apraclonidine. It is worth noting that these eye drops are commercially available in other strengths (cocaine: 2–10%; apraclonidine: 0.5–1.0%) which is likely to affect the size of the pupillary response. Moreover, I chose to routinely measure these effects after 40 min whereas some other studies have preferred to report effects after 60 min. Nevertheless, the effects of these drugs in the normal pupil are broadly in line with what has been described previously. What is striking in this large dataset is the wide range of effect of these drugs; in some cases I even observed miosis after cocaine and mydriasis after apraclonidine.

Some of the factors influencing the effect of these drugs could be identified by further analysis of my data. Both age and pupil size markedly affected the responses to apraclonidine, and I interpret this result as indicating that older patients with smaller pupils have a lower basal sympathetic tone so are less likely to be affected by the alpha-2 actions of this drug. It is interesting that cocaine, working by a different mechanism, was not so clearly affected by basal sympathetic tone. However, the effect of cocaine was strongly influenced by eye color, with the drug having twice as much effect in blue and green eyes than in brown eyes. The explanation for this observation probably lies in the known strong affinity of cocaine for melanin: the more melanized brown iris binds more of the cocaine, reducing its bioavailability at the sympathetic neuro-effector junctions [A similar phenomenon is well known when testing hair specimens for signs of drug abuse, where the test is more sensitive with Africoid hair than with Caucasoid hair; (22)]. For both drugs, the effect on the normal pupil depended on whether the measurements were made in the dark or in bright light. This finding is easy to understand for apraclonidine, since I would expect the alpha2 (miotic) effects to be most apparent when the sympathetic basal tone is increased in the dark. It is less clear why the mydriatic effect of cocaine is 25% greater in the light than in the dark, but I would speculate that it may have something to do with the physical/elastic properties of iris stroma such that a given amount of activation of the dilator muscle will produce more pupil change if starting from a smaller size compared with a larger size.

Although not investigated in this study, I can also identify on theoretical grounds other factors likely to influence the effect of these drugs on the normal pupil. There will be some variation in the drug concentration (“batch effect”) and dose delivered (one drop, several drops, or even no drops if it is an uncooperative patient). The status of the ocular surface plays a vital role in determining drug penetration into the eye; excessive lacrimation dilutes the drug, whereas a compromised corneal epithelium (e.g., in a dry eye) leads to increased drug penetration. The bioavailability of any drug that enters the eye at the neuro-effector junction will be affected by aqueous dynamics and blood flow in the anterior segment of the eye, as well as by drug-binding (as mentioned above for cocaine and melanin). Finally, there will be variation in the basal level of neurotransmitter release and in the availability of adrenergic receptors according to a wide range of genetic and other influences.

Both drugs had a significantly different effect on the pupil in HS eyes compared with normal eyes; cocaine produced on average 1.3 mm less mydriasis, and apraclonidine 1.2 mm more mydriasis, a difference that is easily detected by clinical observation alone without the need for pupillometry or other devices available only to the specialist. However, just as with the observations made in normal pupils, there was a wide range in the drug effects found in HS eyes. At the extreme ends of this range I found cases of HS where the pupil dilated more than 2 mm after cocaine or constricted by up to 0.4 mm after apraclonidine. It is likely that most of this variability in drug effect is due to the same factors identified for normal pupils. In addition, it is possible that some cases were incorrectly classified as HS (the pupillometric and clinical evidence used for this classification cannot be 100% accurate, and there is no other “gold standard” that can be applied for diagnosing HS). Moreover, I chose to routinely measure the drug effect after 40 min, which may be too soon for the test to “turn positive”; there have been a few cases of suspected unilateral HS where the anisocoria only reversed with apraclonidine after a patient has returned home, long after administration of the drops.

In my “real world” dataset, where none of these various influences have been controlled for in either cohort, I have been able to define the 95% limits of the effect of these drugs in the normal eye. Using this definition in suspected cases of HS I can regard any drug effect that lies within these normal limits as a “negative” result (i.e., no evidence of HS), and any result outside these limits as a “positive” test result (HS confirmed). With this approach, cocaine has an estimated sensitivity for detection of HS of only 40%; in effect, this means that if the cocaine test were relied upon as the only means of diagnosing HS then more than half the cases would be missed (“false negatives”) giving false reassurance to both doctor and patient. An alternative approach proposed in a previously published report (13) is to define the test as positive for HS if the anisocoria after cocaine measures ≥0.8 mm; with this definition I estimate the test sensitivity to be rather better at 76%. However, this approach can only be used in cases of unilateral HS and would be of little or no value in my institution which is a tertiary referral center for patients with generalized autonomic failure where HS is usually bilateral.

In comparison to cocaine, apraclonidine seems to be a much more accurate test. I estimate the sensitivity of apraclonidine testing to be around 93% when measured in the dark (and a little less at 76% if measured in bright light). It should be noted that in cases of unilateral HS, the anisocoria does not always reverse; if this is used as the definition of a positive test result then in my series this criterion gives a lower test sensitivity of 75% (whereas applying the cut off defined from my normative data gives a sensitivity of 93% for the same patients). In effect this means that used in isolation (i.e., with no other evidence taken into account) apraclonidine testing can identify almost all cases of HS (false negatives only 7%) and rarely implies HS in a normal eye (false positives only 5%). Compared with other tests used in ophthalmology [for example, intraocular pressure as a screening test for glaucoma (23)], these estimates of sensitivity and specificity are very encouraging and confirm the validity of using this approach even in cases where the signs of oculosympathetic paresis are masked because there is no asymmetry in pupil size or lid position.

Although my data suggest that apraclonidine is a better test for HS than cocaine, there are limitations to its use in clinical practice. Firstly, the abnormal pupil response of HS eyes follows upregulation of alpha1-receptors on the surface of the denervated dilator muscle fibers, a process which takes time and will not be evident immediately after onset of the oculosympathetic paresis. In most cases, for example those associated with tumors or other compressive lesions, this delay until the test turns positive does not matter. However, in the particular instance of internal carotid artery dissection (ICAD), patients may present within hours of onset and it is necessary to make an urgent (same day) diagnosis. Anecdotal case reports have been published of positive apraclonidine test results observed within days or even hours after ICAD (24, 25), but equally there are also reports of false negative results (17). No systematic prospective study has yet been reported to address the question of how long it takes after lesion onset for the sensitivity of the apraclonidine test to achieve a level at which it can be relied on clinically to make management decisions for that patient. In practice I suspect this rarely matters since the clinical presentation of ICAD is so distinctive that all patients are likely to need carotid imaging irrespective of any pharmacological test result. A second and more concerning limitation to apraclonidine testing regards its safety in infants under the age of 1 year. There have been a few reports of adverse systemic reactions to topical administration of this drug—including apnoea, bradycardia, hypotension, somnolence, and lethargy (26) although not all reported studies have encountered problems (18). In some (rare) cases malignant tumors such as neuroblastoma may present with isolated HS in the first year of life, so if there is a strong clinical suspicion and no other reasonable explanation then these patients will probably need a full work-up regardless of any pharmacological confirmation of HS. In my hospital which treats only adults, I have seen no adverse drug reactions to 0.5% apraclonidine after administering this drug to 383 eyes, leading me to conclude that in the adult population this test is completely safe. Finally it should be noted that in this study I have only considered the effects of these drugs on the pupil; apraclonidine also retracts the upper lid (27), but this effect has not been evaluated in the current study and it is not known whether this sign has any diagnostic value for detection of Horner syndrome.

In conclusion, my retrospective data collected over a 14 year observation period shows a wide range of effect of both cocaine and apraclonidine when used as a test for HS. When 95% limits are defined based on the pupil responses I observed in the normal eyes (i.e., giving each drug test a specificity of 95%), cocaine only has a sensitivity of 40% compared with 93% for apraclonidine. In addition, when compared with apraclonidine cocaine is more expensive, less often available and needs special arrangements to be kept securely. On that basis I recommend apraclonidine is now adopted as the “gold standard” pharmacological test for diagnosing HS. Caution may be needed using this test immediately after the onset or in infants under 1 year of age.

## Author Contributions

The author confirms being the sole contributor of this work and has approved it for publication.

### Conflict of Interest Statement

The author declares that the research was conducted in the absence of any commercial or financial relationships that could be construed as a potential conflict of interest.
